# Supplementary material for: α-Lactalbumin mRNA-LNP Evokes an Anti-Tumor Effect Combined with Surgery in Triple-Negative Breast Cancer
Source: Pharmaceutics. 2024 Jul 14;16(7):940. doi: 10.3390/pharmaceutics16070940 (PMC11279974; doi:10.3390/pharmaceutics16070940)
Supplement: Supplementary file 1 [file pharmaceutics-16-00940-s001.zip › Figure S1.pdf]

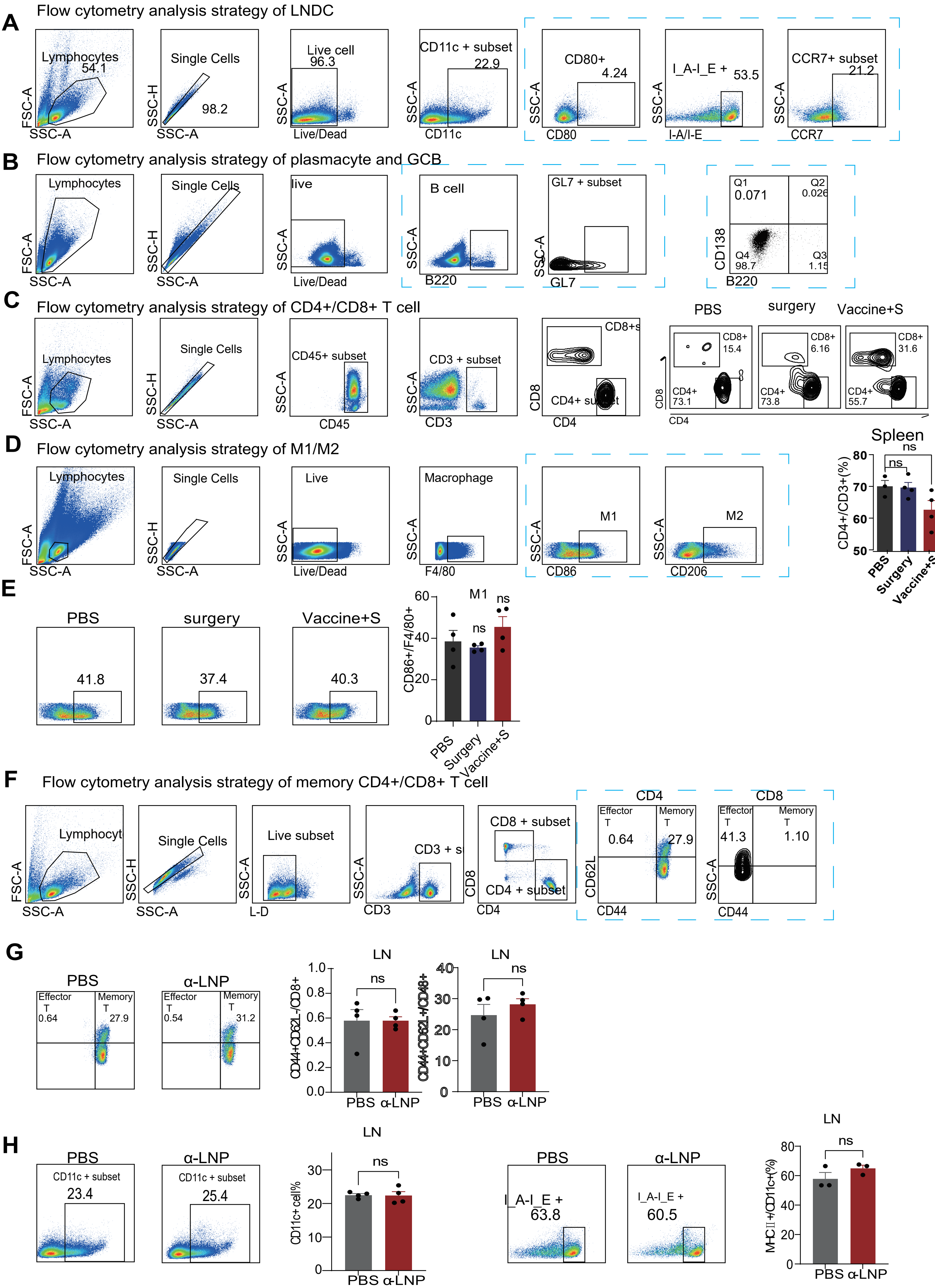
A
Flow cytometry analysis strategy of LNDC
Lymphocytes 54.1
Single Cells 98.2
Live cell 96.3
CD11c + subset 22.9
CD80+ 4.24
I_A-I_E + 53.5
CCR7+ subset 21.2
FSC-A
SSC-A
SSC-H
Live/Dead
CD11c
CD80
I-A/I-E
CCR7
B
Flow cytometry analysis strategy of plasmacyte and GCB
Lymphocytes
Single Cells
live
B cell
GL7 + subset
Q1 0.071
Q2 0.026
Q4 98.7
Q3 1.15
CD138
B220
GL7
C
Flow cytometry analysis strategy of CD4+/CD8+ T cell
CD45+ subset
CD3 + subset
CD45
CD3
CD8
CD4
PBS
surgery
Vaccine+S
CD8+ 15.4
CD4+ 73.1
CD8+ 6.16
CD4+ 73.8
CD8+ 31.6
CD4+ 55.7
D
Flow cytometry analysis strategy of M1/M2
Live
Macrophage
F4/80
M1
M2
CD86
CD206
Spleen
ns
CD4+/CD3+(%)
PBS
Surgery
Vaccine+S
E
41.8
37.4
40.3
CD86+/F4/80+
F
Flow cytometry analysis strategy of memory CD4+/CD8+ T cell
Live subset
CD8 + subset
CD4 + subset
L-D
Effector T 0.64
Memory T 27.9
Effector T 41.3
Memory T 1.10
CD62L
CD44
G
α-LNP
Effector T 0.54
Memory T 31.2
LN
CD44+CD62L-/CD8+
CD44+CD62L+/CD8+
H
CD11c + subset 23.4
CD11c + subset 25.4
CD11c+ cell%
I_A-I_E + 63.8
I_A-I_E + 60.5
MHCⅡ+/CD11c+(%)
