# Supplementary figures and images for: α-Lactalbumin mRNA-LNP Evokes an Anti-Tumor Effect Combined with Surgery in Triple-Negative Breast Cancer
Source: Pharmaceutics. 2024 Jul 14;16(7):940. doi: 10.3390/pharmaceutics16070940 (PMC11279974; doi:10.3390/pharmaceutics16070940)

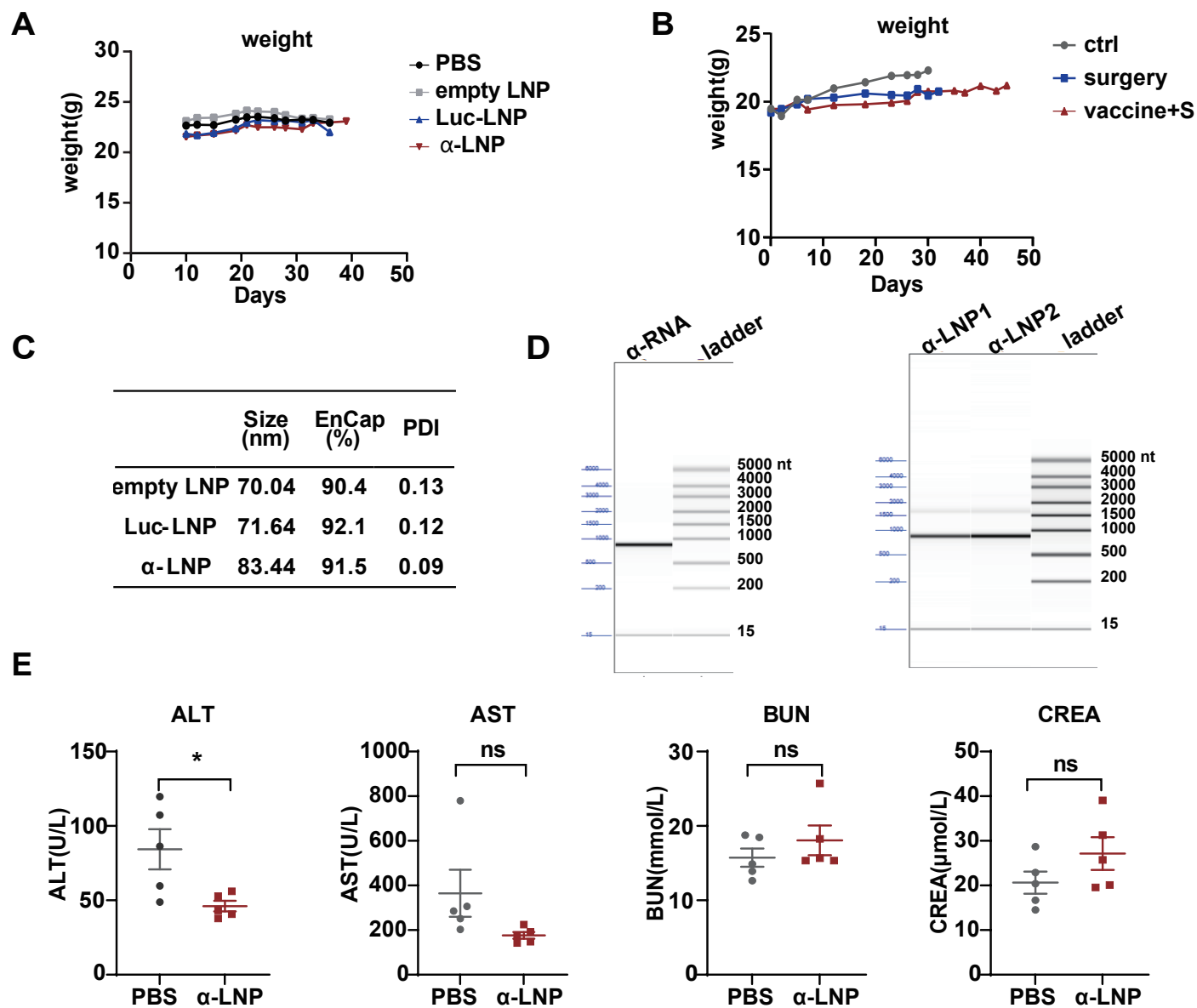

Supplement: Supplementary file 1 [file pharmaceutics-16-00940-s001.zip › Figure S2.pdf]

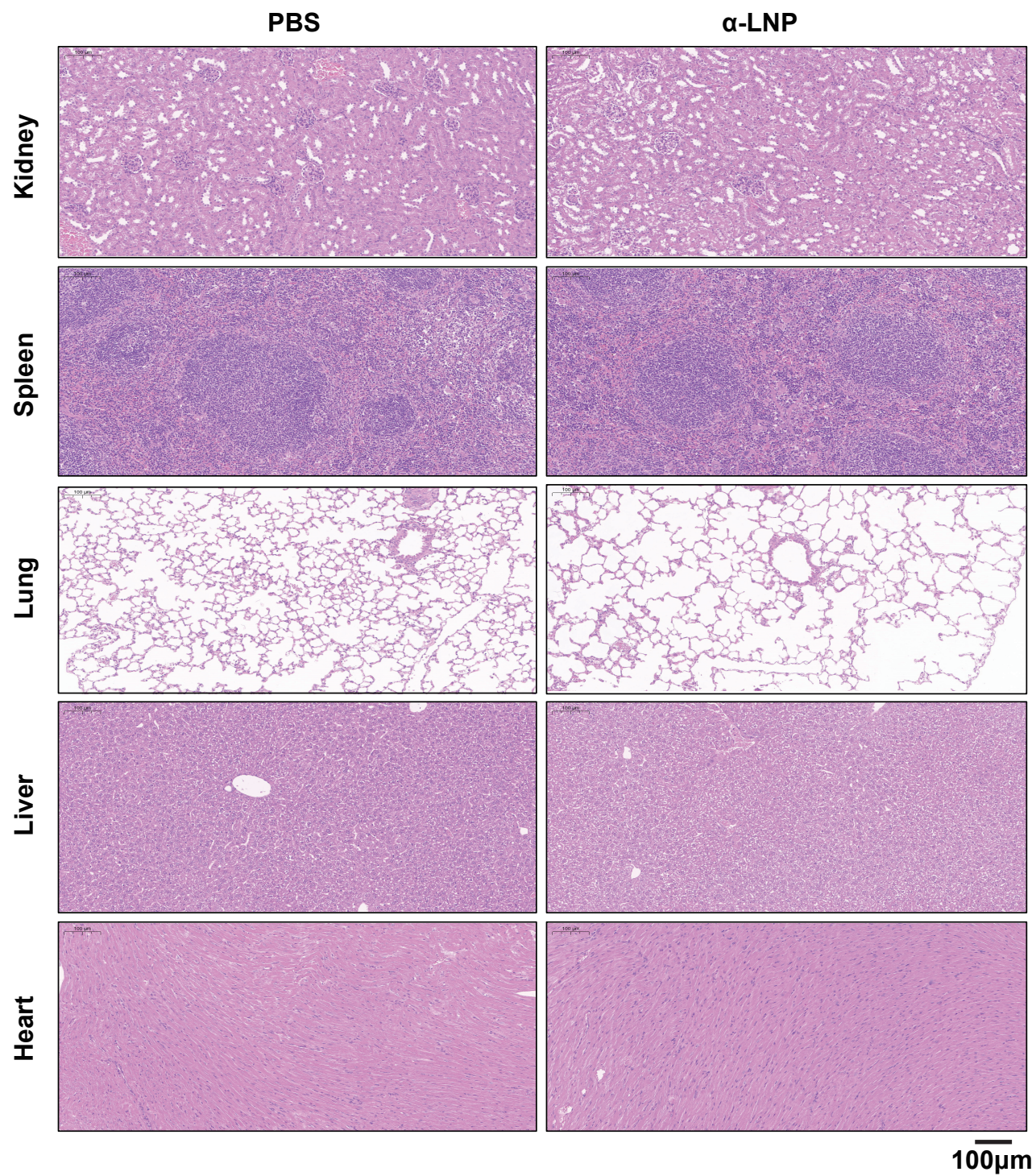

Supplement: Supplementary file 1 [file pharmaceutics-16-00940-s001.zip › Figure S3.pdf]
